# Biological Potential of Carnivorous Plants from Nepenthales

**DOI:** 10.3390/molecules28083639

**Published:** 2023-04-21

**Authors:** Magdalena Wójciak, Marcin Feldo, Piotr Stolarczyk, Bartosz J. Płachno

**Affiliations:** 1 Department of Analytical Chemistry, Medical University of Lublin, Chodzki 4a, 20-093 Lublin, Poland; 2 Chair and Department of Vascular Surgery and Angiology, Medical University of Lublin, 11 Staszica St., 20-081 Lublin, Poland; 3 Department of Botany, Physiology and Plant Protection, Faculty of Biotechnology and Horticulture, University of Agriculture in Kraków, 29 Listopada 54 Ave., 31-425 Cracow, Poland; 4 Department of Plant Cytology and Embryology, Institute of Botany, Faculty of Biology, Jagiellonian University in Kraków, 9 Gronostajowa St., 30-387 Cracow, Poland

**Keywords:** carnivorous plants, biological activity, secondary metabolites, naphthoquinones, polyphenols

## Abstract

Since Charles Darwin and his book carnivorous plants have aroused interest and heated debate. In addition, there is growing interest in this group of plants as a source of secondary metabolites and in the application of their biological activity. The aim of this study was to trace the recent literature in search of the application of extracts obtained from families Droseraceae, Nepenthaceae, and Drosophyllaceae to show their biological potential. The data collected in the review clearly indicate that the studied Nepenthales species have great biological potential in terms of antibacterial, antifungal, antioxidant, anti-inflammatory, and anticancer use. We proposed that further investigations should include: (i) bioactivity-guided investigations of crude plant extract to connect a particular type of action with a specific compound or a group of metabolites; (ii) a search for new bioactive properties of carnivorous plants; (iii) establishment of molecular mechanisms associated with specific activity. Furthermore, further research should be extended to include less explored species, i.e., *Drosophyllum lusitanicum* and especially *Aldrovanda vesiculosa*.

## 1. Introduction

For centuries, plants have always been of great importance for human health and have been used to treat many diseases. Nowadays, plant-derived products or isolated compounds are also of great significance, and the exploration of their biological potential is the subject addressed by numerous research teams. New sources of biomolecules or new directions of action of known compounds are still being sought.

Since Charles Darwin and his book [1], carnivorous plants have aroused interest and heated debate [2,3,4]. Plants evolved various strategies (mycorrhiza, myrmecophily, symbiosis with nitrogen-fixing bacteria) to live in nutrient-poor habitats, and carnivory is an example of such adaptation. Macroelements are absorbed from the bodies of captured organisms; however, carnivorous species also perform photosynthesis and assimilate carbon dioxide using the C3 pathway, thus, can be considered as mixotrophic [5]. When Charles Darwin (1875) published his book about carnivorous plants, he used the word “insectivorous”. However, since then, our knowledge of this ecological group has increased significantly. Although insects are an important prey group, they capture prey ranging from protozoa and various invertebrates to even small vertebrates such tadpoles, fish fry, salamanders, geckos, or, in rare cases, mammals [6,7,8]. In some carnivorous plants, there is a change from carnivory to coprophagy. *Roridula* plants capture insects but have no digestive enzymes, so they use nitrogen from the feces of obligately associated, carnivorous hemipterans [9]. Some *Nepenthes* species developed mutualistic relationships with small mammals for nitrogen supplementation. Animal feces are a source of macroelements [10,11]. Another strategy occurs in *N. ampullaria*, which collects in pitchers the plant-derived materials and benefits from this material utilization [5]. This “vegetarian” trend also occurs in *Utricularia* and *Genlisea*, which capture and digest algae in addition to animal prey [12,13]. Another carnivorous plant strategy to obtain nutrients is cooperation with various organisms (arthropods, fungi, protozoa, and bacteria). Many carnivorous plants with pitcher trap type form small, relatively self-contained ecosystems in traps. Organisms in traps may act as decomposers, breaking down the proteins, fats, and carbohydrates of prey, thus may increase the availability of nutrients for the host plant. The best examples of these inquilines are known in some *Nepenthes* and *Sarracenia* species [14,15]. Similar strategies occur in *Utricularia* and *Genlisea* traps, where various species of bacteria, algae, and protozoa may live and reproduce [16,17,18,19,20].

In recent years, many carnivorous species have gained increased attention due to their multidimensional biological activity and the possibility of their application in pharmacy and medicine. The review by Miclea clearly showed the potential of carnivorous plants from Sarraceniaceae [21]. Similarly, species from the Nepenthaceae and Droseraceae families exhibit diverse biological activities. They have been used in folk medicine to treat various disorders, and recent reports confirm their effectiveness based on scientific investigations. Plants of both families are a rich source of secondary metabolites representing phenolics (flavonoids including anthocyanins, phenolic acids, and their derivatives), naphthoquinones, and volatile organic compounds [22,23,24,25,26], and the role of these components in the growth, development, and physiology of plants has previously been described in detail by Hatcher et al. [27]. The aim of our work was to summarize the current state of knowledge on the biological effectiveness of Droseraceae and Nepenthaceae, especially since both crude extracts and isolated components of these families have been intensively studied in terms of antimicrobial, anticancer, and anti-inflammatory action [28,29,30]. It should also be noted that some *Dionaea*, *Drosera*, and *Nepenthes* species are common in cultivation and easy to propagate and introduce into in vitro cultures. Thus, their biomass is relatively easily available, and these plants may be a valuable model for biological and phytochemical studies.

The review discusses the biological activity of plants, and the provided information can help to outline the directions for further investigation of carnivorous species. A literature survey was carried out using Scopus, PubMed, Web of Science, and google scholar databases. In the investigation following combination of terms was used: “*Aldrovanda*” or “*Nepenthes*” or “*Drosera*” or “*Dionaea*” or “*Drosophyllum*” and “activity” or “action”. The search covered titles, abstracts, and keywords. The lists of all retrieved articles (ca 1700 papers) were reviewed, taking into account the inclusion criteria (full-text availability, English language) and exclusion criteria (conference papers, reviews, the subject area not connected with biological science, e.g., environmental science, engineering, chemistry, physics and so on). From the selected 279 papers, further identification of potentially relevant studies was performed by abstract screening.

## 2. Occurrence and Taxonomy

**Figure 1:**
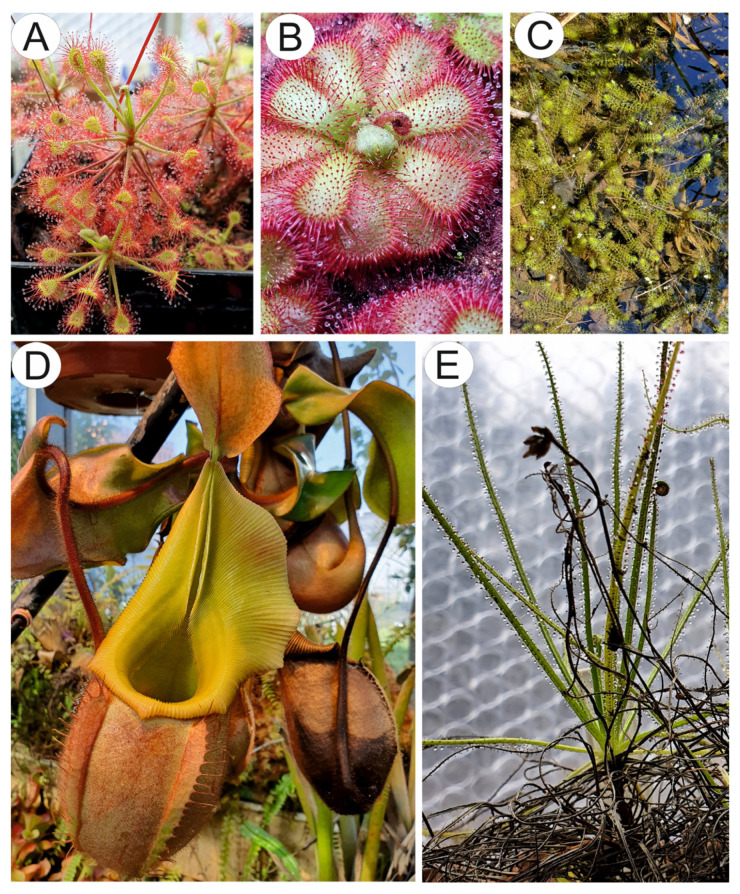
Example of species from carnivorous Nepenthales: (**A**). *Drosera madagascariensis* DC., (**B**). *Drosera admirabilis* Debbert, (**C**). *Aldrovanda vesiculosa* L. at a replacement site near Třeboň in the Czech Republic. (**D**). *Nepenthes veitchii* Hook.f. in the carnivorous plant collection of Dr. Krzysztof Banaś (University of Gdańsk). (**E**). *Drosophyllum lusitanicum* (L.) Link.

Nepenthales is an ancient (Late Cretaceous: Cenomanian and Gondwanan origin) taxonomically diverse order comprising five families: Droseraceae, Nepenthaceae, Drosophyllaceae, Dioncophyllaceae, and Ancistrocladaceae. However, the members of this monophyletic group differ from their sister core-Caryophyllales, in terms of the occurrence of acetogenic naphtho- and anthraquinones and the absence of betalains [4,31,32]. The Droseraceae family consists of three carnivorous genera: *Drosera* (Figure 1A,B), *Dionaea,* and *Aldrovanda* (Figure 1C). The *Drosera* genus is species rich about 250 species, but new species are still described (Figure 1C) [33], and is nearly cosmopolitan. *Drosera* are herbaceous carnivorous plants with leaves with glandular emergences [34] forming active adhesive traps [35]. The *Dionaea* genus is monotypic and contains one extant species of *Dionaea muscipula*, which is paleoendemic, with restricted distribution (to the coastal plain of North and South Carolina on the eastern seaboard of the United States) [34]. The *Aldrovanda* genus comprises several extinct species and one extant species, *Aldrovanda vesiculosa,* which used to be widespread in the Old World and in various climatic zones; however, now it is rare and endangered [36,37]. Both *Dionaea* and *Aldrovanda* have highly mobile snap traps characterized by different mechanics [38].

The family Nepenthaceae is related to Droseraceae and is represented by only one genus *Nepenthes* (Figure 1D), which is widespread in the Australasian tropics with the highest diversity in the islands of Borneo, Sumatra, and the southern Philippines. This genus comprises more than 160 species [31,39], and still new species are being discovered [40]. *Nepenthes* plants produce pitchers to catch prey. However, some highly specialized species also use other nutritional sources such as mammal and bird feces or plant litter [10,11,41].

The Drosophyllaceae family comprises one species *Drosophyllum lusitanicum* (Figure 1E), which is a carnivorous subshrub with leaves with glandular emergences that form passive adhesive traps. It is endemic to the western Iberian Peninsula and northern Morocco, where it grows in fire-prone Mediterranean heathlands [42,43,44].

The Dioncophyllaceae family comprises one carnivorous genus, *Triphyophyllum,* and two non-carnivorous genera (*Dioncophyllum* and *Habropetalum*). The *Triphyophyllum* genus contains one species, *Triphyophyllum peltatum*, which occurs in tropical West Africa [31]. *T. peltatum* produces carnivorous leaves with emergences during its juvenile phase [45].

Figure 1 shows some examples of carnivorous species of Nepenthales.

## 3. Biological Activity

### 3.1. Antibacterial, Antiviral, and Antifungal Activity

Antibacterial and antifungal properties are the most widely explored biological activity of Droseraceae plants. In recent years, many in vitro studies have confirmed their efficacy against various pathogens that are resistant and susceptible to antibiotics. Different species have been intensively studied, and extracts have been prepared from both fresh and dried plant material using solvents with diverse polarity. The following parameters are used to describe the antibacterial effect: MIC—minimal inhibitory concentration (the lowest concentration that shows no visible growth), MBC/MFC—minimum bactericidal/fungicidal concentration (the lowest concentration that reduces viability by 99.9%), or zone of inhibition—ZOI (the radius of the circle around the discs with an antimicrobial agent, in which bacteria growth is inhibited).

Many reports show that plants from the Droseraceae and Nepenthaceae families are active against fungal pathogens and Gram-positive (G(+)) bacteria; however, their potential against Gram-negative (G(−)) bacteria is significantly lower [46,47]. This is not surprising because G(−) bacteria are, in general, more resistant to antimicrobials than G(+) bacteria due to the presence of a lipopolysaccharide-rich outer membrane, which provides additional protection against external agents [48]. For example, analyses of extracts from some Brazilian *Drosera* species (*Dr. communis*, *Dr. montana* var. *montana*, *Dr. brevifolia*, *Dr. villosa* var. *graomogolensis*, and *Dr. villosa* var. *villosa*) demonstrated that they were effective against *Staphylococcus aureus* and *Candida albicans*; in turn, *P. aeruginosa*, *E. coli*, and S. *choleraesuis* were resistant to all these extracts [46]. A similar observation was made for *Dionaea muscipula* extracts against food-related pathogenic and putrefactive bacteria. The inhibitory effect against G(+): *B. cereus*, *B. subtilis*, *M. luteus, S. aureus*, and *S. faecalis* was approximately two times higher than in the case of G(−): *E. coli, P. aeruginosa, S. enteritidis, S. typhimurium,* and *S. marcescens* [49]. On the other hand, no tendency was observed in the case of *Dr. peltata* against oral bacteria, and the activity of chloroform extract against G(−) anaerobes: *Preotella oris, P. buccae*, and *P. intermedia* was higher (or comparable) than against some *Streptococcus strains* [50].

Many studies have evidenced the antimicrobial potential of Droseraceae species. *Dr. rotundifolia* extracts were active against various pathogens, including *Bacillus thuringiensis, Clostridium perfringens, Listeria monocytogenes, E. coli, Salmonella enterica subsp. enterica*, and *Yersinia enterocolitica* [51,52]. In addition, ethanol extracts from the field and lab-grown *Dr. rotundifolia* showed significant antiviral activity. They were found to be able to protect adenocarcinomic human alveolar basal epithelial cells (A549) against enteroviruses CVA9 and CVB3, responsible for many acute and chronic infections [53]. In another study, methanol extract and its fractions (petroleum ether, chloroform, ethyl acetate, n-butanol, and water residues) from *Dr. peltata* var. *lunata* were tested against *Rhizopus oryzae*, *Aspergillus flavus, A. niger, A. oryzae*, and *Penicillium citrinum*. The petroleum ether fraction was the most active (MIC = 5.86–46.88 µg/mL, MFC = 23.44–93.75 µg/mL), and the effect was related to plumbagin [54]. Grevenstuk et al. reported a growth-inhibiting effect of *Dr. intermedia* extracts and isolated plumbagin against yeasts and filamentous fungal strains responsible for food deterioration [55]. Antibacterial activity was also exhibited by some compounds from the bulbs of *Dr. magna*. Naringenin-6-C-β-d-glucopyranoside showed the broadest spectrum of activity and, at a concentration of 32 µg/mL, displayed a 100% inhibitory effect against methicillin-resistant *S. aureus* (MRSA), *E. coli*, *K. pneumoniae*, *Acinetobacter baumanii, P. aeruginosa, Candida albicans*, and *Cryptococcus neoformans*. Hirsutrin was also effective against most of the microorganisms mentioned above, except *C. albicans*, and was only partially active toward *P. aeruginosa* (79% of inhibition). In turn, hydroxydroserone and plumbagin acted against *Cryptococcus neoformans* and MRSA [56].

The aforementioned extracts were prepared using leaves or whole plants; however, the other parts of plants also showed an antibacterial effect. Aqueous, ethanol, and methanol extracts of thick roots, open flowers, and hair of *Dr. spatulata* var. *bakoensis* were evaluated against respiratory tract infectious microbes, including *S. aureus, K. pneumoniae, S. pneumoniae*, and *A. niger*. Among the extracts tested, ethanol was the most active, with MIC in the range of 0.35–0.50 mg/mL, 0.3–0.45 mg/mL, 0.45–0.55 mg/mL, and 0.5–0.6 mg/mL, respectively [57].

The impact on biofilm creation is another important direction of action in the context of antibacterial activity. The multilayered cellular coating formed by polysaccharides and proteins (biofilm) of bacterial strains protects against drugs and increases the resistance of pathogens to treatment [58]. The biofilm inhibitory effect of *Dr. rotundifolia*, *Dr. intermedia*, and four commercial sundew products against multidrug-resistant *E. coli* strains was examined by Gerschler et al. They demonstrated that plant extracts were more effective than commercial products (minimum biofilm inhibitory concentrations MBIC were 35 µg/mL and 75–140 µg/mL, respectively), and 2”-*O*-galloyl hyperoside was the most potent inhibitor of the isolated compounds (MBIC: 38 µg/mL). It should also be mentioned that plumbagin and 7-methyl juglone were responsible for the antibacterial effect, and the flavonoids were not active [52].

Although there are many reports on the antibacterial activity of Droseraceae species, little is known about Nepenthaceae. Only a few papers have been published on this topic. Ethanol extract from dried *N. gracilis* was effective against *Bacillus subtilis* and *E. coli* with an inhibition zone of 19 and 17 mm for the leaf, and 11 and 9 mm for the pitcher, respectively (control: 16 and 14 mm, respectively) [59], and hexane extract inhibited the growth of some fungi [60]. In turn, acetone extract from *N*. cv. Miranda showed antibacterial activity against *S. aureus*, *P. aeruginosa*, and *E. coli* [61] and against *K. pneumoniae* [62], *N. mirabilis* was effective against *S. aureus* [63], and extract from *N. bicalcarata* leaves was active against *S. aureus*, *B. subtilis, B. spizizenii*, and non-filamentous fungi (*S. cerevisiae* and *C. albicans*) [64]. Furthermore, an antifungal effect against human pathogens, including *Candida* and *Aspergillus* spp., was exhibited by chitin-induced pitcher liquid as well as isolated naphthoquinones from *N. khasiana* [65].

In turn, almost nothing is known about the activity of Dioncophyllaceae and Drosophyllaceae plants, and only one study was published. Investigations of the antibacterial/antifungal activity of *Drosophyllum lusitanicum* against nine bacterial and 11 yeast strains showed that it was effective against Gram (+) bacteria (*S. aureus*, *S. epidermidis*, *E. faecalis, S. pyogenes*), with the exception of *S. pneumoniae*: ZOI = 17.67–43 mm (15.6 to 250 µg/mL) and the tested yeast strains: ZOI = 23.67–42.23 mm and MIC values in the range of 31–63 µg/mL. In contrast, the growth of Gram-negative bacteria (*P. aeruginosa*, *E. coli*, *E. sakazakii*) was only slightly inhibited by the plant extract [66].

**Figure 2:**
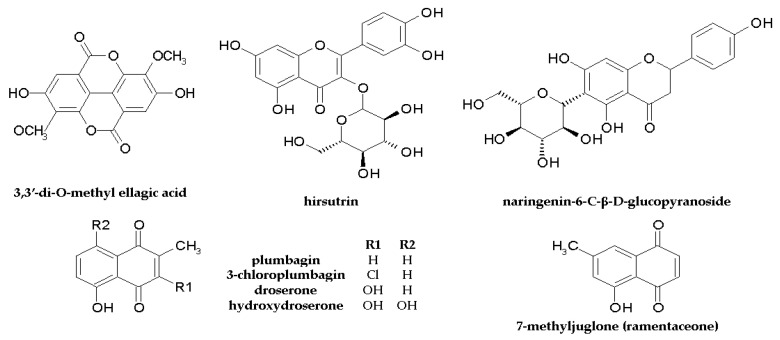
Chemical structure of components with antibacterial and antifungal activity found in Nepenthales.

More details on the anti-pathogenic activities of extracts from Droseraceae, Drosophyllaceae, and Nepenthaceae species and isolated compounds, including MIC/MBC values, are given in Table 1 and Table 2. The chemical structures of the components are shown in Figure 2.

Different approaches were used to enhance the antibacterial activity of carnivorous plants. For example, it has been evidenced that the combination of plant extracts with silver nanoparticles (AgNPs) increased the bactericidal activity of *Dr. gigantea, Di. muscipula, Dr. binata, Dr. indica,* and *Dr. spatulata* against *P. aeruginosa*, *S. aureus*, *C. albicans* fungi, and plant pathogens *Pectobacterium atrosepticum, P. parmentieri*, and *Dickeya dadantii* [67,71]. It also enhanced the effectiveness of *Dr. binata* extract against multidrug-resistant clinical strains of *S. aureus* [68], as well as *Dr. spatulata* against *E. coli* [72]. Moreover, a synergistic effect with AgNPs was also observed for isolated naphthoquinones, i.e., plumbagin and 3-chloroplumbagin [67,68,73].

It has also been shown that supplementation of medium with various factors may increase the antibacterial activity of in vitro cultivated plants, as this procedure has an impact on the content of secondary metabolites from the naphthoquinone class. Such an increase was observed for *Di. muscipula* after elicitation with *Cronobacter sakazaki* lysate [74] as well as for *Dr. capensis* and *Di. muscipula* subjected to *Agrobacterim rhizogenes* lysate, jasmonic acid, nitrogen deficiency, L-phenylalanine, and trans-cinnamic acid [69]. Furthermore, the antibacterial potential of *D. muscipula* against *S. aureus, E. faecalis, E. coli*, and *P. aeruginosa* increased significantly after the genetic transformation of tissues using *Rhizobium rhizogenes* strains [70].

The antibacterial and antifungal activity of carnivorous plants is considered to be mainly related to the presence of naphthoquinones, such as plumbagin and its isomer ramentaceone; however, the other components of the plants act synergistically and enhance their effectiveness. It has been demonstrated that naphthoquinones are effective inhibitors of nucleic acid synthesis in pathogen cells. Moreover, plumbagin may induce apoptosis by inactivating topoisomerase II through the generation of free radicals [47,75]. In turn, flavonoids exhibit anti-biofilm activity, which has great significance in pathogen infection [52], and some flavonoids isolated from carnivorous plants have significant anti-pathogen activity as well [56]; therefore, the antimicrobial activity is a result of the synergistic action of various components.

It should also be mentioned that the proteins found in pitcher fluid also exhibit a significant antibacterial/antifungal effect. They are pathogenesis-related proteins protecting carnivorous plants during the digestion of prey [76,77,78,79].

### 3.2. Cytotoxic Activity

**Figure 3:**
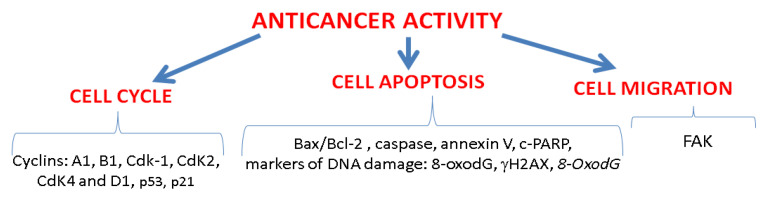
Main molecular mechanisms and the investigated parameters associated with anticancer activity studied in carnivorous plants from Nepenthales.

Anticancer activity is another explored direction of action of Droseraceae and Nepenthaceae plants, and some reports have been published on this topic. The investigations were based on both in vitro studies on cell lines and in vivo assays using animal models. Basic investigations included cytotoxicity assays based on cellular metabolic activity tests (MTT—mitochondrial dehydrogenase activity, LDH—lactate dehydrogenase activity) or permeability of cell membrane tests (TB—trypan blue). Additionally, some molecular mechanisms associated with anticancer activity have also been studied, focusing on (i) the cell cycle (cell cycle regulatory proteins: cyclin A1, cyclin B1, and Cdk-1), (ii) the apoptotic pathway (Bax/Bcl-2 ratio, caspase, annexin V, markers of DNA damage: 8-Hydroxy-2-Deoxyguanosine, γH2AX) and (iii) cell migration (FAK protein—focal adhesion kinase) (Figure 3). All these mechanisms are of great significance in the development of anticancer therapies [80].

Ethanol and aqueous extracts from whole *Dr. indica* plants showed cytotoxicity against Dalton’s Ascitic Lymphoma (DAL) and Ehrlich Ascitic Carcinoma (EAC) cell lines and remarkably increased the percentage of dead cancer cells at 250 µg/mL (90% and 86%, respectively, in DAL cells and 89% and 80%, respectively, in EAC) [81]. Furthermore, the activity was confirmed in an in vivo study in a murine model with induced Dalton’s lymphoma ascites (DLA). After a 14-day treatment with the extracts (250 and 500 mg/kg orally), normalization of body weight, viable tumor cells, and hematological parameters were observed, which were altered in the untreated DLA group. This was accompanied by increased caspase-3 activity and decreased DNA, RNA, and protein content in the treated group, suggesting an increase in apoptosis [82]. Similar effects were exerted by *Dr. burmannii* extracts in an in vivo study in mice with induced EAC [83]. Furthermore, *Dr. peltata* and *Dr. indica* had a modulatory effect on the development of metabolic syndrome in tumor-bearing mice and stabilized the tumor-induced serum hormones, blood glucose, and lipid profile [84,85]. In turn, in an in vitro study, hexane, ethyl acetate, and 1-butanol fractions were obtained by fractionation of extract (10% MeOH/water) from fresh leaves of *Di. muscipula* and were found to be cytotoxic against P388 murine lymphocytic leukemia [86].

*Dr. burmannii* methanol/water (7:3) extract was cytotoxic against breast cancer (MCF-7) with IC_50_ 120.9 µg/mL; however, it had no impact on the other cancer lines tested, i.e., lung (A549), cervical (HeLa), liver (HepG2), and brain (U87) cells (IC_50_ in the range of 1062–1577 µg/mL) and on the control normal fibroblast cell line (WI-38 IC_50_ 1389 µg/mL). It downregulated the expression of cell cycle regulatory proteins (cyclin A1, B1, and cyclin-dependent kinase Cdk-1) and arrested MCF-7 cells in the G2/M phase but did not affect the G1/S regulatory proteins CdK2, CdK4, and D1. Furthermore, it activated tumor-suppressor proteins p53 and cyclin-dependent kinase inhibitor p21 [87]. p53-p21-RB signaling is an important cell cycle regulator, and p53 can induce cell cycle arrest, as it downregulates many cell cycle genes [88]. It was also evidenced that *Dr. burmannii* extract induced apoptosis through the disturbance of the Bax (apoptotic)/Bcl-2 (anti-apoptotic) protein ratio and the activation of the caspase cascade [87]. Bax/Bak are pore-forming proteins at the mitochondrial outer membrane, and their expression leads to an increase in the permeability of the mitochondrial membrane and the release of cytochrome c to the cytosol, where it binds to the cytosolic proteins Apaf-1 and procaspase-9 and forms an apoptosome. This is followed by the activation of caspases responsible for the proteolytic degradation of cellular structures and, thus, the induction of apoptosis [89,90]. The degradation of poly (ADP-ribose)polymerase (PARP) to its cleaved form (c-PARP) was also observed [87]. PARP is involved in various cellular processes, such as DNA repair, cell division, differentiation, and programmed cell death [91].

Further detailed insight into the mechanism of anticancer action was provided by investigation of a few *Nepenthes* species. In an in vitro study, it was found that an ethyl acetate fraction of methanol extract from *N. thorellii* x (*ventricosa* x *maxima*) exerted an anti-proliferative effect against breast cancer (MCF7 and SKBR3) [92] and oral cancer (Ca9-22, CAL 27, OECM-1, and HSC-3) [93] and exhibited antileukemic activity against three leukemia cell lines, including acute promyelocytic HL-60, chronic myelogenous K-562, and T-cell acute lymphocytic MOLT-4 (IC_50_ in the range of 3.68–3.85 µg/mL in a 24-h assay) [94]. It was established that the mechanism of action was associated with the induction of oxidative stress, as an increase in the ROS level and mitochondrial superoxide (MitoSOX), mitochondrial membrane depolarization, and enhanced mRNA expression of genes involved in the modulation of oxidative stress, i.e., nuclear factor erythroid 2-like 2 (NFE2L2), catalase (CAT), thioredoxin (TXN), heme oxygenase 1 (HMOX1), and NAD(P)H quinone dehydrogenase 1 (NQO1), were observed after treatment with *Nepenthes* extract. As a result of oxidative stress, increased apoptosis, and DNA damage were noted, which was evidenced by an increase in caspases 3/7, γH2AX, and 8-hydroxy-2-deoxyguanosine markers of DNA damage, and expression of c-PARP and cleaved caspase-3 (c-Cas 3) [92,93,94].

A similar mechanism was reported for the ethyl acetate fractions of methanol extract from *N. adrianii* x *clipeata, N. ventricosa* x *maxima*, and *N. ventricosa* x *sibuyanensis.* In an in vitro study, they exhibited cytotoxicity against various types of oral cancer (Ca9-22, CAL 27, OECM-1, SCC9, and HSC-3) through induction of oxidative stress-mediated apoptosis and DNA damage [95,96,97]. Furthermore, it was established that the cytotoxic effect was enhanced in a combination of the extract with ultraviolet-C (UVC) irradiation. The proliferation of Ca9-22 cells was decreased to 57% compared to the individual treatments with the extract (5 µg/mL) and a low dose of UVC (12 J/m2) (80%). No cytotoxicity against normal oral HGF-1 cells was observed [98].

Only one study was devoted to the anticancer activity of Drosophyllaceae plants. Water, methanol, and hexane leaf extracts of *Drosophyllum lusitanicum* were studied in terms of their anti-proliferative, cytotoxic, and apoptogenic properties against human cervical adenocarcinoma [99]. It has been shown that the hexane extract was the most active, followed by the methanol extract, and induced cell cycle arrest in the G2/M phase and apoptosis [99]. On the other hand, there are no reports on direct anticancer activity of *Triphyophyllum peltatum*; however, Bringmann et al. found that some dioncoquinones (which belong to naphthylisoquinoline alkaloids) occurring in stress-induced plant cell cultures showed antitumor activity against lymphoma (DOHH-2 and SU-DHL-4) and multiple myeloma (INA-6) in an in vitro study [100].

A few studies have shown that carnivorous plant extracts can enhance the activity of known anticancer compounds. *N.* cv. Miranda-leaf-acetone combined with 5-fluorouracil synergistically increased apoptosis of pulmonary adenocarcinoma (PC-9), carcinoma (4T1), and murine melanoma (B16F10) cells [62], and a combination of ethyl acetate extract from *N. ventricosa* x *maxima* with cisplatin can enhance the cytotoxicity against oral cancer cells (Ca9-22) [96].

It is worth mentioning that the extracts and isolated compounds were less or non-toxic against normal cell lines used as reference. Detailed information on the cytotoxic activity of Droseraceae and Nepenthaceae is summarized in Table 3.

Many reports indicate that the anticancer activity of carnivorous plants is associated with naphthoquinones, whose cytotoxicity against various types of cancer lines is well documented. In particular, the anticancer potential of plumbagin has recently been extensively studied [101,102,103,104,105,106]. It was found that plumbagin affects the various signaling pathways involved in cancer cell proliferation, survival, invasion, and metastasis through suppression of some signaling molecules such as nuclear factor-kappaB (NF-κB) and signal transducer and activator of transcription 3 (Stat3) [107,108]. In vitro, investigation showed that plumbagin generates ROS and regulates the PI3K/Akt and MAPK signaling pathways to promote apoptosis and autophagy [109]. Furthermore, in silico study revealed that plumbagin can bind to cancer signaling proteins, namely PI3Kγ, AKT1/PKBα, Bcl-2, NF-κB, and Stat3, which play a key role in the pathogenesis of cancer [110]. It also can form H-bonds with PARP [111]. Some review papers have been published to summarize the latest findings on this topic [112,113,114,115,116]. Therefore, here, we focused only on naphthoquinones isolated from Droseraceae and Nepenthaceae plants.

As reported by Kawiak et al., 3-chloroplumbagin induces apoptosis as it disturbs the ratio of apoptotic (Bax, Bak) to anti-apoptotic (Bcl-2, Mcl-1) proteins [117]. Furthermore, it has been shown that the compound affects MAP kinase signaling and reduces the levels of phosphorylated MEK and ERK induced by EGF, which increases the sensitivity of cancer cells to the induction of apoptosis. These results suggest that ChPL induces apoptosis via both MAP kinase signaling and the mitochondria-mediated pathway [117].

Similarly, it was found that the cytotoxicity of ramentaceone against human promyelocytic leukemia was mediated via triggering apoptosis through the mitochondria-mediated pathway and ROS generation, and several features characteristic for induction of the process, i.e., a decrease in the mitochondrial transmembrane potential, the Bcl-2/Bax, Bak ratio, an increase in the cytochrome c level, and the activity of caspase 3, were observed [118].

The anticancer effects of plumbagin from *N. alata* against MCF-7 breast cancer cells were also related to an increased intracellular ROS level resulting in the induction of apoptosis via a p53-dependent pathway (↑p53 and p21). Plumbagin arrested the cell cycle in G2/M (↓cyclin B1, no effect on cyclin A). An increase in c-PARP and Bax protein and a decrease in Bcl-2 were also noted [119]. It was also found that, through ROS induction, plumbagin inhibits topoisomerase II and stabilizes the topoisomerase II−DNA cleavable complex, thus contributing to DNA damage [75]. Moreover, plumbagin turned out to be a potent inhibitor of dihydroorotase and, therefore, may decrease the biosynthesis of pyrimidines essential for the rapid growth and proliferation of cancer cells [120]. Plumbagin also exerted an inhibitory effect on allantoinase [62] and dihydropyrimidinase [121] involved in the utilization of nitrogen in purine-derived compounds. These metal-dependent enzymes belong to the cyclic amidohydrolase family and catalyze the hydrolysis of the cyclic amide bond in the metabolism of purines and pyrimidines; therefore, they are important targets for anticancer therapy.

The anticancer activity of plumbagin was confirmed in an in vivo study using an MCF-7 tumor xenograft model developed with BALB/c nude mice. Plumbagin injected at 2 mg/kg body weight for 4 weeks markedly inhibited the increase in tumor volume and decreased tumor weight [119].

Examples of the cytotoxicity of naphthoquinones isolated from Droseraceae and Nepenthaceae species against different types of cancers are shown in Table 4.

It should be mentioned that the anticancer activity of *Drosera* species can also be attributed to the pentacyclic triterpene 3-O-acetylaleuritolic acid (3-O-AAA), which was found in *Dr. spatulata* [123] and *Dr. villosa* [46]. The compound exerted a growth inhibitory effect on HeLa (cervical cancer), HT-29 (human colorectal adenocarcinoma), and MCF7 (human breast cancer) cell lines with IC_50_ 2.26 μM, 3.43 μM, and 4.23 μM, respectively, after 24 h of treatment (control: non-tumorigenic breast cells IC_50_ = 53 μM). It moderately affected colony formation, inhibited cell migration (the scratch assay) and cell adhesion (↓FAK protein), and contributed to autophagy induction (modulation of autophagy-related proteins including mTOR and beclin1 proteins) [123].

On the other hand, the cytotoxicity of some carnivorous species may be connected with flavonoid compounds. Quercetin 3-O-(6″-n-butyl-d-glucuronide), which was one of the main active components of the fraction from *N. thorellii* [92], showed a cytotoxic effect against liver cancer HepG2 and breast cancer MCF7 cells [124]. Moreover, there are many reports on the anticancer properties of flavonoids [125,126,127,128], and this allows an assumption that different compounds can act synergistically.

**Figure 4:**
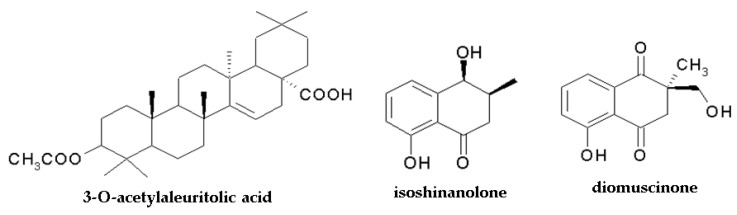
Chemical structure of components with anticancer activity found in Nepenthales. The structure of plumbagin, 3-chloroplumbagin, and ramentaceone is shown in Figure 2.

The structures of compounds with confirmed anticancer activity found in Nepenthaceae and Droseraceae are presented in Figure 2 and Figure 4.

### 3.3. Anti-Inflammatory Effects

Some reports describe the anti-inflammatory activity of Nepenthaceae and Droseraceae. The HET-CAM assay (hen’s eggs test–chorioallantoic membrane) revealed a strong anti-inflammatory property of ethanol and aqueous extracts from aerial parts of *Dr. rotundifolia* and ethanol extract from *Dr. madagascariensis* at the doses of 500 μg of extracts/pellet; the anti-inflammatory effect was stronger than that exerted by 50 μg of hydrocortisone/pellet (control) [129]. The anti-inflammatory activity was also noted for 70% ethanol extract from dried aerial parts of *Dr. madagascariensis* [130] and *Dr. rotundifolia* [131] based on the human neutrophil elastase activity assay, and it was found that the activity was related to flavonoids, in particular quercetin derivatives [132]. In turn, *Dr. burmannii* methanol/water extract (30–80 µg/mL) downregulated the NO and TNF-α level increase in RAW 264.7 (murine macrophage) cells through bacterial lipopolysaccharide (LPS)-induced inflammation by modulation of the mRNA expression of iNOS and COX-2. It also suppressed LPS-stimulated intracellular ROS production [87].

In another study, the anti-inflammatory effect of ethanol/water extract from some *Drosera* species on human mast cells (HMC-1) induced with T cell membrane activated by PMA (phorbol 12-myristate 13-acetate) was investigated [133]. Activation of T cell membranes (aTc-m) leads to the degranulation of mast cells and the release of inflammatory mediators, including cytokines; therefore, mast cells are involved in allergic and inflammatory reactions [134]. It was found that *Dr. rotundifolia* and *Dr. tokaiensis* at 200 µg/mL suppressed aTc-m-induced expression of TNF-α, Granzyme B (GZMB), interleukine-1 (IL1β), and intracellular adhesion molecule-1 (ICAM-1) in HMC-1 cells. In turn, no effect was observed for *Dr. spatulata* [133]. The analysis of the phenolic composition of the extracts revealed that hyperoside, isoquercitrin, myricetin, quercetin, and ellagic acid were the main components, with the content in the range of 2.81–4.02%, 0.40–0.71%, 0.22–0.49%, 0.41–0.53%, and 0.17–2.63%, respectively [133].

On the other hand, Thao et al., who investigated the anti-inflammatory activity of methanolic extract from *N. mirabilis* as well as its components in LPS-stimulated bone marrow-derived dendritic cells, found that all the isolated compounds, i.e., naphthalene derivatives, lignans, triterpenes, and flavonoids, decreased the production of pro-inflammatory cytokines IL-12p40 and IL-6; however, they had slight or no effects on TNF-α production. 2-methoxy-7-methyljuglone followed by nepenthoside B were the most potent inhibitors with IC_50_ 0.17 (IL-12p40), 0.46 (IL-6), 8.28 (TNF-α) µM and 1.17 (IL-12 p40), 2.15 (IL-6), and 21.05 (TNF-α) µM, respectively [135].

Anti-inflammatory activity assays of Droseraceae and Nepenthaceae plants are summarized in Table 5.

It is commonly believed that the anti-inflammatory effect of carnivorous plants is associated with flavonoids, which can inhibit pathological processes associated with inflammation, including proteolytic damage of tissues induced by neutrophil elastase and the release of lysosomal enzymes. They can also modulate the expression of pro-inflammatory cytokines [136,137]. For example, quercetin, isoquercitrin, and hyperoside, the main flavonoids found in *Dr. rotundifolia*, inhibited human neutrophil elastase with IC_50_ values of 0.8, 0.7, and 0.9 µg/mL, respectively, but no effect was exerted by plumbagin and junglon [131]. In turn, Fukushima et al. observed no correlation between the contents of phenolic compounds and anti-inflammatory activity [133]; therefore, further investigation is needed to clarify this issue.

### 3.4. Antioxidant Activity

Maintenance of the redox balance is crucial for the proper function of cells. Reactive oxygen species (ROS), normally produced as part of aerobic metabolism, play an essential role in the regulation of many physiological processes, e.g., they contribute to defense against microorganisms. However, an excessive ROS level may lead to damage to cell macromolecules, including DNA, protein, and membrane lipids, and oxidative stress is involved in aging processes and the pathogenesis of various disorders. On the other hand, increased ROS levels trigger apoptosis of cancer cells; therefore, ROS inducers have a high potential in anticancer therapy [138,139].

The antioxidant activity of Droseraceae and Nepenthaceae species was extensively investigated using in vitro (e.g., DPPH, ABTS, FRAP, ORAC, antioxidant enzyme activity) and in vivo assays. Generally, carnivorous plants contain two types of metabolites with different antioxidant effects; thus, the activity of extracts depends on the type of the extrahent used. Methanol or ethanol extracts with various water addition levels contain many polyphenolic compounds with strong antioxidant activity [53,64,74,81,140]. In turn, less polar extracts (acetone, ethyl acetate, chloroform) rich in naphthoquinones have a lower capability to scavenge ROS in in vitro assays [47,99]. Detailed analysis of the antioxidant capacity of compounds isolated from *N. mirabilis* (ORAC assay and reduction of Cu^2+^ to Cu^+^) showed that quercetin and kaempferol glycosides were significantly more active than naphthoquinones [141]. On the other hand, naphthoquinones stimulate ROS production and contribute to ROS-mediated cell apoptosis, which is beneficial in anticancer therapy. This was evidenced by an increased ROS level in many types of cancer cell lines (Table 2) treated with acetone or ethyl acetate extracts from *N. thorelii* [92,93,94], *N. adrianii* [95], and *N. ventricosa* [96,97].

It should also be noted that some extracts or plant-derived compounds exert ambiguous antioxidant effects. They exhibit activity in free radical scavenging tests, such as ABTS or DPPH; however, in assays carried out on cell line models, they strongly generate ROS. The impact of exogenous antioxidants is often concentration-dependent. They may alleviate oxidative stress at low concentrations and stimulate ROS generation at higher amounts [138].

### 3.5. Other Types of Activities

In addition to the most widely explored anti-pathogenic, anticancer, anti-inflammatory, and antioxidant properties mentioned above, a few reports have described other biological effects for *Drosera* and *Nepenthes* species.

#### 3.5.1. Impact on Respiratory Tract

According to ethnomedicine, some carnivorous plants, e.g., *Drosera* species, are useful in the treatment of respiratory tract disorders, including chronic bronchitis, asthma, and cough. A few recent reports have verified this type of activity.

The effect of a low concentration of *Dr. rotundifolia* ethanolic extract containing phenolic compounds (ellagic acid, quercetin, isoquercitrin, and/or hyperoside) on the human bronchial epithelial cell line (16HBE) was evaluated. After 24 h of incubation, a ca 6.9% increase in cell viability was noted. Furthermore, the extract stimulated the cell functions by regulating the expression of dozens of genes involved in various cellular processes. For example, *Dr. rotundifolia* extract increased the expression of specific epidermal growth factors, which play a positive role in the regulation of cell survival, cell proliferation, and wound healing. It also enhanced the expression of proteins related to xenobiotic detoxification and cytokines; therefore, it stimulated self-repair systems impaired in respiratory tract diseases [142].

In another in vitro study, the effects on airway smooth muscle (ASM) and ciliary beat frequency (CBF) were investigated using tracheal slices of C57BL/6N mice [143]. It was found that *Dr. rotundifolia* extract (90% ethanol) and an aqueous fraction increased the CBF, which improved mucociliary clearance. Furthermore, the ethanol–water extract and the aqueous fraction had an antispasmodic effect against acetylcholine-induced contractions, and the water fraction abrogated potassium ions-induced contraction. Quercetin, 2″-O-galloylhyperoside, and hyperoside flavonoids were found to be responsible for an impact on smooth muscle, as they accelerated CBF, exerted an inhibitory effect on phosphodiesterases [143], enzymes involved in airway dilatation, vascular and airway smooth muscle contraction, and remodeling, and participated in the regulation of inflammation [144].

#### 3.5.2. Anti-Osteoporotic Activity

*N. mirabilis* was found to be a source of polyphenols and naphthoquinones with anti-osteoporotic activity and high potential in the prevention and treatment of osteoporosis. Among the isolated compounds, pinoresiol-4-O-b-D-glucopyranoside, nepenthosides B [145], plumbagin, cis-isoshinanolone, quercetin 3-O-b-D-glucuronide, and kaempferol-3-O-a-L-rhamnoside [141] in an in vitro assay exerted the strongest inhibitory effect on the differentiation of osteoclasts, which are responsible for bone resorption and play an important role in the formation of the bone matrix. Tartrate-resistant acid phosphatase (TRAP was used) as a phenotypic marker in the study because TRAP expression is associated with the induction of osteoclast differentiation [146]. The most active compounds, namely plumbagin and kaempferol-3-O-a-L-rhamnoside, at 10.0 mM inhibited TRAP to 14.73% and 35.98%, respectively, compared to the control (RANκL-induced RAW 264.7 cells) [141].

#### 3.5.3. Antiparasitic Activity

The antimalarial activity of chloroform extract from *N. thorelii* roots and isolated compounds was evaluated by Likhitwitayawuid et al. using *Plasmodium falciparum* [147]. The plant extract exhibited a significant inhibitory effect with IC_50_ 10 µg/mL, and plumbagin was found to be the most active of the investigated components (IC_50_ = 0.27 µM); therefore, it is considered the main component responsible for the antimalarial activity of *N. thorelii*. Only a weak antiplasmodial effect was exhibited by the other isolated components, namely 2-methylnaphthazarin, octadecyl caffeate, isoshinanolone, and droserone (IC_50_ in the range of 5.79–22.06 µM).

Furthermore, hydroxydroserone isolated from *Dr. magna* bulbs showed anthelmintic activity against fourth-stage larvae of the blood-feeding parasitic nematode *Hemonchus contortus* and inhibited larval motility by 27% at 100 μM after 72 h [56].

The antimalarial activity was also reported for *Triphyophyllum peltatum*. Chloroform extracts from root and stem bark showed antiplasmodial activity with IC_50_ values of 103 and 279 ng/mL for *P. berghei* and 53 and 76 ng/mL for *P. falciparum*, respectively [148]. Presumably, the effect was related to the presence of naphthylisoquinoline alkaloids, as some of the isolated compounds, namely 5’-O-demethyldioncophylline A, dioncophylline C, dioncopeltine A, dioncopeltine A, and dioncophylline A and B, exhibited significant activity against both protozoa [148,149,150]. Interestingly, dioncophylline A also showed larvicidal activity against the mosquito *Anopheles stephensi*, capable of transmitting both *P. falciparum* and *P. vivax* [151] and was effective against *Aedes aegypti*—a known vector of several viruses, including yellow fever, dengue, chikungunya and Zika viruses [152]. On the other hand, betulinic acid, representing lupane-type triterpenes, isolated from *T. peltatum* also exhibited moderate to good in vitro antimalarial activity against asexual erythrocytic stages of *P. falciparum* [153].

It should also be mentioned that dioncoquinones found in *T. peltatum* showed promising antiprotozoal bioactivities against *Leishmania*, *Trypanosoma* [154], and *Babesia canis* [155].

#### 3.5.4. Antiaging Activity

The antiaging effect of some *Drosera* species was evidenced by Tominaga et al. based on the impact of 80% MeOH extracts from aboveground parts of *Dr. rotundifolia*, *Dr. tokaiensis*, *Dr. spatulata*, and *Dr. peltata* on advanced glycation end-products (AGEs) including N^ω^-(carboxymethyl)arginine (CMA) and N^ε^-(carboxymethyl)lysine (CML). The accumulation of AGEs is correlated with aging and the development of age-related diseases, such as diabetes and arteriosclerosis. It was found that crude extracts significantly inhibited the formation of CMA and CML, and the effect was related to purified components, such as ellagic acid, 3,3′-di-O-methylellagic acid 4′-glucoside, myricitrine, and quercimelin [156].

#### 3.5.5. Hepatoprotective Activity

70% methanolic extract from dried *Dr. burmannii* displayed significant hepatoprotective activity in an in vivo study in iron-overloaded Swiss albino mice, normalized the serum markers increased after hepatocellular injury, including ALAT, ASAT, ALP, and bilirubin, and enhanced the levels of liver antioxidants. The effect was a result of strong iron chelation activity [140].

#### 3.5.6. Antiepileptic Activity

In an in vivo assay, Hema et al. found that 90% ethanolic extract from *Dr. burmannii* at the dose of 500 mg/kg body weight showed antiepileptic activity, delayed the onset of convulsions, and decreased the duration of seizures induced with pentylenetetrazole (PTZ) in mice administered one hour before PTZ [157].

#### 3.5.7. Antidiabetic Effect

Methanolic leaf extract of *N. bicalcarata* exhibited antidiabetic activity in an in vivo study; administered orally at a dose of 300 mg/kg body weight for 6 weeks, it significantly reduced the blood glucose level in diabetic rats [64].

In turn, *N. mirabilis* exhibited α-glucosidase and α-amylase inhibitory activity. Both enzymes regulate blood glucose levels, and the inhibition of these enzymes can delay the digestion of carbohydrates, resulting in a decrease in the rate of glucose absorption. Therefore, the extracts have the potential for use against type 2 diabetes [158]. The effect may be associated with plumbagin because in silico study revealed that it may interact with α glucosidase, an enzyme involved in the metabolism of carbohydrates and insulin production [159].

#### 3.5.8. Support of Celiac Disease Treatment

Proteolytic components in the *N.* x *ventrata* pitcher can support the treatment of celiac disease, i.e., a chronic autoimmune disorder triggered by gluten proteins commonly occurring in grain products. It has been shown that, due to the combined activity of prolyl endoprotease and a non-canonical aspartic protease, the fluid from pit-fall traps had a potent gluten detoxification capacity. Therefore, supplementation with enzymes derived from *Nepenthes* plants can help to avoid the induction of immune responses by reducing peptide size [160].

#### 3.5.9. Antifertility Activity

An in vivo study revealed significant antifertility activity of 90% ethanol and aqueous extracts from *Dr. burmannii* [161]. Both extracts administered orally at a dose of 250 or 450 mg/kg body weight caused loss of implantation ability in female rats with regular estrous cycles. The alcohol extract decreased the percentage of implantation to 78.74% and 85.23%, respectively. In turn, the aqueous extract reduced the implantation to 67.22% and 83.66%. The antifertility activity may be related to the plumbagin presented in the ethanol extract [162]; however, no plumbagin was found in the water extract. Thus, further investigation is needed.

The less widely studied types of activity of carnivorous Nepenthales plants are summarized in Table 6.

#### 3.5.10. Activity against Plant Pathogens and Pests

Hexane extract from the leaf of *N. ventricosa* x *maxima* effectively inhibited plant pathogenic fungi, including *P. capsic, R*. *stolonifer* var. *stolonifera*, *A. alternata, A. niger, B. oryzae, F. oxysporum, R. solani*, and *S. sclerotiorumand* with MIC values ranging from 7.2 to 43.7 μg/mL [163].

Furthermore, pitcher and leaf tissues of *N.* x *ventrata* (hybrid of *N. alata* and *N. ventricosa*) exerted a growth inhibitory effect, and larvicidal activities against insect herbivores *Spodoptera littoralis*, and the action was related to naphthoquinones. It was found that the plumbagin concentration necessary for 50% growth inhibition of larvae was determined to be 226.5 µg/g diet [164]. Plumbagin isolated from *Dr. muscipula* leaves showed antifeedant activity against *Spodoptera litura* [86,165] and had significant insecticidal activity against adults of *Musca domestica* (LD_50_ 20 µg/fly) and reduced their longevity, fertility, and natality [166]. Insecticidal activity against *Liriomyza trifolii* through contact application was also exhibited by hexane extracts from *Drosophyllum lusitanicum* leaves (at a concentration of 100 mg/mL, it caused 100% mortality after 1 d of treatment) [167]. Hexane and water extracts also showed toxicity against lettuce and wheat and significantly inhibited seed germination [168].

Moreover, recombinant chitinase derived from *Drosera* spp. showed antifungal potential and suppressed the growth of some plant pathogens, including *Fusarium poae*, *Trichoderma viride*, *Alternaria solani* [169] and *Parastagonospora nodorum* [170].

In turn, naphthylisoquinoline alkaloids, namely dioncophylline A and 5’-O-demethyl-8-O-methyl-7-epi-dioncophylline A, isolated from *T. peltatum* exhibited a molluscicidal effect with LD_100_ values of 20 and 40 ppm, respectively [171].

All these data indicate that compounds derived from carnivorous plants may be promising agents helpful in the control of plant diseases and pest management.

## 4. Possibility of Cultivation

Most Nepenthales plants have limited natural habitats, and some of them are strictly protected [172]. This raises the problem of obtaining plant material with sufficient biomass for phytochemical and biological investigations and for the isolation of active molecules. Therefore, alternative ways for the production of biomass are constantly being developed, e.g., field or greenhouse cultivation, in vivo propagation, or micropropagation and suspension culture [122,173,174,175,176,177,178,179,180] which may increase the possibility of large-scale applications in pharmacy and medicine.

For example, vegetative propagation was successfully applied to increase plant material of various *Drosera* spp. [53,122,181,182,183,184,185,186,187], *Dionaea muscipula* [69,188,189,190], *Nepenthes* spp [191,192,193] and *Drosophyllum lusitanicum* [177,178,194]. In this technique, explants from parent plants are cultivated in a growth medium, and 3 to 12 plants can be obtained from one explant after 6–8 weeks. In the cultivation process, various solidified and liquid media, e.g., Murashige and Skoog (MS), half-strength MS, Lindemann, Vacin and Went, Fast, etc., can be applied. The medium is usually supplemented with various growth regulators, including auxins: 2,4-dichlorophenoxyacetic acid, naphthylacetic acid, indoleacetic acid, and cytokinins: kinetin, 6-benzylaminopurine, and gibberellins. The pH of the growth medium is also an important factor affecting plant growth and development [184,185]. Obviously, there is no universal medium composition that would be optimal for in vitro propagation of different species, and optimization of experimental conditions is usually needed to obtain a high reproduction rate.

Furthermore, genetic transformation and elicitation using different growth regulators (e.g., methyl jasmonate, yeast extract, and chitosan) have been studied to increase the production of active metabolites and, in consequence, the biological potential of Droseraceae and Nepenthaceae, and it was found that carnivorous plants are susceptible to elicitation [69,74,184,193,195]. For instance, the beneficial effect on naphthoquinones content in the roots of *Dr. burmanii* and *Dr. indica* was observed for yeast extract [186,196]. Furthermore, chitosan, salicylic acid, and methyl jasmonate were found to be elicitors for plumbagin in in vitro culture of *Dr. indica* [196], and it was evidenced that production of plumbagin in shoots of *Dr. peltata* may be stimulated by 6-benzyladenine [184]. In turn, L-phenylalanine and trans-cinnamic acid enhanced the production of flavonoids (quercetin and myricetin) in *Dr. capensis* and *Di. muscipula* [69] and a combination of biotic elicitator (*Cronobacter sakazakii* lysate) with hydromechanical stress increased the level of polyphenolic compounds in *Di. muscipula* [74]. In recent years, it was also found that the color of the light source or microwave radiation used during cultivation may influence the level of metabolites in carnivorous plants [197,198,199,200].

## 5. Conclusions and Future Prospects

There is a growing global interest in plant-derived products containing many bioactive compounds with various biological activities and providing health benefits.

The data collected in the review clearly indicate that species from the genera *Nepenthes*, *Drosera*, and *Dionaea* have great biological potential, and their activity is a subject of interest for many researchers. Intensive investigation is being conducted in terms of antibacterial, antifungal, antioxidant, anti-inflammatory, and anticancer properties of extracts, isolated fractions, and pure compounds.

Among them, the greatest attention has recently been paid to cytotoxicity against different types of cancer. It has been evidenced that some *Nepenthes* and *Drosera* species show anticancer activity against lymphocytic leukemia [86,94], oral [93,95,96,97], and breast [87,92] cancer cell lines. This effect was mainly attributed to naphthoquinones, and the mechanism of action is related to increased generation of intracellular ROS resulting in apoptosis induction through MAP kinase signaling and p53-dependent and mitochondria-mediated pathways [117,118,119]. Naphthoquinones also are responsible for antibacterial, antiviral, and antifungal activities and may be of great importance in the context of the search for a new alternative to traditional antibiotics against microbial infections due to the increased prevalence of microbial resistance [54,55,56,60].

Interestingly, it has also been evidenced that the extract or isolated compounds combined with known cytostatic or antibacterial agents may enhance anticancer and bactericidal activity. Such a synergistic effect was observed, e.g., for *N.* cv. Miranda extract combined with 5-fluorouracil against pulmonary adenocarcinoma and murine melanoma [62], ethyl acetate extract from *N. ventricosa* x *maxima* with cisplatin against oral cancer cells [96], and 7-methyljuglone combined with antituberculous drugs against *Mycobacterium tuberculosis* [201,202]. In addition, the combination of *Drosera* spp. with silver nanoparticles (AgNPs) seems to be a promising direction for further investigation, as it increased the activity against multidrug-resistant bacterial strains, fungi, and plant pathogens [67,68,71,72]. A synergistic effect with AgNPs was also exhibited by isolated naphthoquinones, i.e., plumbagin and 3-chloroplumbagin [67,68,73].

In turn, polyphenols are related to anti-inflammatory [129,130,131,133], antiosteoporotic [141,145], antispasmodic [131,131,143], and antiaging [156] activities and to the beneficial effect on the respiratory tract [142,143].

Our paper summarizes the current state of knowledge on the biological potential of the Nepenthaceae and Droseracea families and clearly shows that detailed biochemical characterization of the species may lead to the discovery of new therapeutic agents. In addition, extracts may be an alternative to synthetic insecticides for pest and plant disease management in agriculture, as they inhibit the activity of plant pathogenic fungi and insect herbivores [163,164,165,166,169,170].

This overview can be used as a starting point for further research that should be developed and focused in the future on (i) bioactivity-guided investigations of crude plant extracts to connect the particular type of action with a specific compound or a group of metabolites, (ii) a search for new bioactive properties of carnivorous plants, and (iii) establishment of molecular mechanisms associated with the specific activity. Moreover, further investigations should be extended to include less commonly explored species. For example, still little is known of *Aldrovanda* and *Drosophyllum*, and researchers have so far focused mainly on the biogeography or morphology of these genera [7,203,204,205,206,207]. To date, only a few reports have been published on their biological activity [66] and phytochemistry [208,209,210,211,212].

Furthermore, efforts should be increased to elaborate cost-effective ways for obtaining plant material, including improvement of micropropagation protocols, genetic transformation, and in vitro cultures with different growth regulators to increase the availability of the plant material. In addition, the testing of different elicitors and cultivation conditions should be continued to enhance the production of biologically active compounds for pharmaceutical and medical purposes.

## Figures and Tables

**Table 1:** Antibacterial and antifungal activity of extracts from Droseraceae, Drosophyllaceae, and Nepenthaceae species.

Species	Extract	Antibacterial/Antifungal Effect	Ref
*Dr. aliciae*	methanol, chloroform	*E. faecalis* MBC: 125–150 mg FW/mL; *S. aureus* MBC: 25–75 mg FW/mL; *E. coli* MBC: 50–100 mg FW/mL; *K. pneumoniae* MBC: 125, >150 mg FW/mL; *P. aeruginosa* MBC:125, >150 mg FW/mL	[47]
*Dr. peltata* var. *lunata*	petroleum ether fraction (PFE) from methanol extract	*R. oryzae* MIC: 23.44; MFC: 93.75 µg/mL; *A. flavus* MIC: 11.72, MFC: 23.44 µg/mL; *A. niger* MIC: 23.44, MFC: 46.88 µg/mL; *A. oryzae* MIC: 5.86, MFC: 23.44 µg/mL; *P. citrinum* MIC: 46.88 MFC: 93.75 µg/mL	[54]
*Dr. peltata*	chloroform	*S. mutans* MIC: 31.25; *S. sobrinus* MIC: 15.62 µg/mL; *S. rattus* MIC: 125; *S. cricetus* MIC: 62.5 µg/mL; *S. sanguis* MIC: 125; *S. milleri* MIC: 125; *S. mitis* MIC: 250 µg/mL; *S. constellatus* MIC: 125; *S. oralis* MIC: 125 µg/mL; *S. salivarius* MIC: 250 *Prevotella oris* MIC: 125 µg/mL; *P. buccae* MIC: 62.5; *P. intermedia* MIC: 62.5 µg/mL	[50]
*Dr. gigantea*	tetrahydrofuran	*P. aeruginosa* BMC 80 mg FW/mL	[67]
*Dr. intermedia*	n-hexane	*A. fumigatus, A. flavus*: MIC: 15.63 μg/mL; *A. niger, A. parasiticus, P. expansum* MIC: 31.25 μg/mL; *Z. bailii, P. membranaefaciens* MIC: 7.80 μg/mL; *S. cerevisiae* MIC: 15.60 μg/mL; *D. hansenii* MIC: 3.90 μg/mL	[55]
*Dr. binata*	chloroform	*S. aureus* MBC: 16 mg DW/mL	[68]
*Dr. capensis*	methanol (m), chloroform (ch)	*E. faecalis* MBC: 125 (ch), 150, >150 (m) mg FW/mL; *S. aureus* MBC: 50 (ch), 75–100 (m) mg FW/mL; *K. pneumoniae* MBC: >150 (ch,m) mgFW/mL; *P. aeruginosa* MBC: >150 (ch, m) mgFW/mL	[69]
*Dr. rotundifolia*	ethanol	*B. thuringiensis, C. perfringens, L. monocytogenes* *E. coli, S. enterica, Y. enterocolitica* MIC: 25 μg DW/mL	[51]
*Dr. intermedia*	methanol	*E. coli* MIC: 367–700 μg/mL	[52]
*Dr. spatulata* var. *bakoensis* root (r), flower (f), hair (h).	ethanol	*S. aureus* MIC: 0.45 (r), 0.3 (f), 0.4 (h) mg dr. ex/mL; *K. pneumoniae* MIC: 0.55 (r), 0.45 (f, h) mg dr. ex/mL; *S. pneumoniae* MIC: 0.5 (r), 0.35 (f), 0.4 (h) mg dr. ex/mL; *A. niger* MIC*:* 0.6 (r), 0.5 (f), 0.65 (h) mg dr. ex/mL	[57]
*Di. muscipula*	methanol (m), chloroform (ch)	*E. faecalis* MBC:75, 100 (ch), 75 (m) mg FW/mL; *S. aureus* MBC: ≤25 (ch, m) mg FW/mL; *K. pneumoniae* MBC: 100, 125 (ch), 75 (m) mg FW/mL; *P. aeruginosa* MBC: >150 (ch), 100, 125 (m) mg FW/mL	[69]
*Di. muscipula*	tetrahydrofuran	*P. aeruginosa* BMC: 160 mg FW/mL	[67]
*Di. muscipula*	tetrahydrofuran	*S. aureus* MIC: 167, BMC: 500 μg DW/mL; *E. faecalis* MIC: 667, BMC: 1250 μg DW/mL; *E. coli* MIC: 500, BMC: 1250 μg DW/mL; *P. aeruginosa* MIC: 1250, BMC: 1250 μg DW/mL	[70]
*Drosophyllum lusitanicum*	hexane	Bacteria: *S. aureus* MIC: 31 µg/mL *S. epidermidis* MIC: 15.6 µg/mL *E. faecalis* MIC: 250 µg/mL*, S. pyogenes* MIC: 125 µg/mL Fungi: *C. albicans, C. catenulate, Trichosporon beigelii* MIC: 31 µg/mL; *C. famata, C. guilliermondi, Yarrowia lipolytica Y, T. mucoides, C. neoformans* MIC: 63 µg/mL	[66]
*N. khasiana*	chitin-induced pitcher liquid	*C. albicans* MIC: 2.5, MFC: 12.5 mg DW/mL; *C. krusei* MIC: 7.3, MFC: 14.5 mg DW/mL *C. glabrata* MIC: 14.5, MFC: 14.5 mg DW/mL; *A. fumigatus* MIC: 3.8, MFC: 14.5 mg DW/mL; *A. flavus* MIC: 1.9, MFC: 1.9 mg DW/mL; *A. niger* MIC: 1.9, MFC: 7.3 mg DW/mL	[65]
*N. bicalcarata*	methanol	*S. aureus, B. subtilis, B. spizizenii, C. albicans* MIC: 256; *S. cerevisiae* MIC: 1024 (μg/mL)	[64]
*N. gracilis*	hexane	*C. albicans, I. orientalis, T. mentagrophytes:* MIC 20 µg/mL (MFC 20 µg/mL); *C. parapsilosis, C. neoformans:* MIC 20 µg/mL (MFC 160 µg/mL); *A. brasiliensis* MIC 40–80 µg/mL (MFC 160 µg/mL)	[60]

MIC—minimal inhibitory concentration (the lowest concentration that shows no visible growth); MBC—minimum bactericidal concentration (the lowest concentration that reduces viability by ≥99.9%); MFC—minimal fungicidal concentration; FW—fresh weight; DW—dry weight.

**Table 2:** Antibacterial and antifungal activity of isolated components from the Droseraceae and Nepenthaceae families.

Compound	Species	Antibacterial/Antifungal Effect	Ref
ramentaceone	*Dr. aliciae*	*E. faecalis* MBC: 0.1 mg/mL; *S. aureus* MBC: 0.05 mg/mL; *E. coli* MBC: 0.08–0.165 mg/mL; *K. pneumoniae* MBC: >0.4 mg/mL; *P. aeruginosa* MBC: >0.4 mg/mL	[47]
plumbagin	*Dr. peltata* var. *lunata*	*R. oryzae* MIC: 5, MFC: 20 µg/mL; *Aspergillus flavus* MIC: 5 MFC 20 µg/mL; *A. niger* MIC: 5 MFC: 20 µg/mL; *A. oryzae* MIC: 0.625, MFC: 2.5 µg/mL; *P. citrinum* MIC: 0.625, MFC: 0.625 µg/mL	[54]
plumbagin	*Dr. intermedia*	*A. fumigatus* MIC: 0.08 μg/mL; *A. flavus* MIC: 0.98 μg/mL; *A. niger, A. parasiticus, P. expansum* MIC: 1.95 μg/mL; *Z. bailii* MIC: 2.00 μg/mL; *S. cerevisiae* MIC: 7.80 μg/mL; *D. hansenii, P. membranaefaciens*: MIC 3.90 μg/mL	[55]
plumbagin 3-chloroplumbagin 3,3′-di-O-methyl ellagic acid droserone	*Dr. binata*	*S. aureus* MBC: 16 µg/mL; *S. aureus* MBC: 32 µg/mL; *S. aureus* MBC: >128 µg/mL; *S. aureus* MBC: >128 µg/mL;	[68]
naringenin-6-C-β-D -glucopyranoside	*Dr. magna*	*S. aureus, E. coli, K. pneumoniae, A. baumanii, P. aeruginosa, C. albicans, C. neoformans:* 100% inhibition at 32 µg/mL	[56]
hirsutrin	*Dr. magna*	*S. aureus, E. coli, K. pneumoniae, A. baumanii, C. neoformans:* 100% inhibition at 32 µg/mL*; P. aeruginosa:* 79% inhibition at 32 µg/mL	[56]
hydroxydroserone	*Dr. magna*	*C. albicans:* 98% inhibition at 8 μg/mL; *C. neoformans:* 100% inhibition at 4 μg/mL,	[56]
plumbagin	*Dr. magna*	*S. aureus:* 96% inhibition at <0.2 μg/mL *C. neoformans:* 100% inhibition at 8 μg/mL,	[56]
plumbagin	*N. gracilis*	*C. albicans, I. orientalis* MIC: 2 µg/mL (MFC: 2 µg/mL); *T. mentagrophytes* MIC: 2 µg/mL (MFC: 4 µg/mL); *C. parapsilosis* MIC: 8 µg/mL (MFC: 16 µg/mL); *C. neoformans* MIC: 4 µg/mL (MFC: 8 µg/mL); *A. brasiliensis* MIC: 31 µg/mL (MFC: 63 µg/mL)	[60]
7-methyl juglone	*Dr. rotundifolia*	*E. coli* MIC: 250–333 µg/mL	[52]
plumbagin	*Dr. rotundifolia*	*E. coli* MIC: 104–208 µg/mL	[52]

MIC—minimal inhibitory concentration; MBC—minimum bactericidal; MFC—minimal fungicidal concentration.

**Table 3:** Cytotoxic activity of Droseraceae and Nepenthaceae extracts.

Species/Extract	Model/Reference Line	Effect	Ref
*Dr. indica*/water (w), ethanol (e)	Dalton’s Ascitic Lymphoma (DAL), Ehrlich Ascitic Carcinoma (EAC)	250 µg/mL ↑ dead cells ca 90% (e), 86% (w) 250 µg/mL ↑ dead cells ca 89% (e), 80% (w)	[81]
*Dr. indica*/water, ethanol	Mice with induced Dalton’s lymphoma ascites (DLA)	14-day treatment (250, 500 mg/kg orally): normalization of body weight and blood parameters, ↓ viable tumor cells, ↑ 3-caspase, ↓ DNA, ↓ RNA, ↓ total protein	[82]
*Dr. burmannii*/water, ethanol	Mice with induced Ehrlich Ascitic Carcinoma (EAC)	14-day treatment (250, 500 mg/kg orally): normalization of body weight and blood parameters, ↓ viable tumor cells	[83]
*Dr. burmannii*/70% methanol	breast cancer (MCF-7)/normal fibroblast cell (WI-38)	↓ viability: IC_50_ 120.9 µg/mL 200 μg/mL: arrested cells in the G2/M phase (↓ cyclins A1, B2, CdK1; ↑ p21, ↑p53) ↑ apoptosis (↑ annexin V, ↑ Bax/Bcl-2, ↑ caspases 9 and 3, ↓ PARP)	[87]
*Di. muscipula*/fractions from 10% methanolic extract	murine lymphocytic leukemia (P388)	IC_50_: 0.01 mg/mL hexane, 6.3 mg/mL ethyl acetate, 15.8 mg/mL butanol fraction	[86]
*Drosophyllum lusitanicum/*hexane (h), methanol (m), water (w)	human cervical adenocarcinoma (HeLa)	IC_50_ = 2.14 μg/mL (h); IC_50_ = 50.98 μg/mL (m); IC_50_ = 719.53 μg/mL (w) ↑ apoptosis (↑ subG1)	[99]
*N.* cv. Miranda/acetone	human pulmonary adenocarcinoma (PC-9), 4T1 mammary carcinoma, B16F10 melanoma	150 µg/mL: 98% death rates inhibition of cell migration	[62]
*N. adrianii* x *clipeata*/ethyl acetate fraction from methanol extract	oral cancer (CAL 27, OECM-1, Ca9-22, HSC-3, SCC9)/normal oral cells (HGF-1)	↓ viability: IC_50_: 8, 11, 12, 14, 17 μg/mL ↑ ROS: ↑ MitoSOX, depletion of MMP ↑ apoptosis (↑ annexin V, ↑ c-PARP) ↑ DNA damage (↑ γH2AX)	[95]
*N. ventricosa* x *maxima*/ethyl acetate fraction from methanol extract	oral cancer (Ca9-22, CAL 27)/normal oral cells (HGF-1)	↓ viability: IC_50_: 11, 12 μg/mL ↑ apoptosis (↑ subG1, ↑ annexin V, ↑ pancaspase) ↑ ROS: ↑MitoSOX, depletion of MMP ↑ DNA damage (↑ 8-oxodG)	[96]
*N. ventricosa* x *sibuyanensis/*ethyl acetate fraction from methanol extract	oral cancer (Ca9-22, CAL 27, SCC9)/normal oral cells (HGF-1)	↓ viability: IC_50_: 25, 20, 32 μg/mL ↑ apoptosis (↑ subG1, ↑ annexin V, ↑ pancaspase) ↑ ROS: ↑ MitoSOX, depletion of MMP ↑ DNA damage (↑ 8-oxodG)	[97]
*N. thorellii* x (*ventricosa* x *maxima*)/ethyl acetate fraction from methanol extract	human breast cancer (MCF7 and SKBR3)/human breast normal cells (M10)	↓ viability: IC_50_ 10 and 15 μg/mL ↑ apoptosis (↑ subG1, ↑ annexin V) ↑ ROS: ↑ GSH, ↑ MitoSOX, depletion of MMP DNA damage (↑ γH2AX, ↑ 8-OxodG)	[92]
*N. thorellii* x (*ventricosa* x *maxima*)/ethyl acetate fraction from methanol extract	oral cancer (Ca9-22, CAL 27, OECM-1, HSC-3)/normal oral cells (HGF-1)	↓ viability: IC_50_ 9.27, 11.05, 13.2, 24 μg/mL ↑ apoptosis (↑ subG1, ↑ annexin V, ↑ pancaspase) ↑ ROS: ↑ MitoSOX, depeletion of MMP ↑ expression of antioxidant genes DNA damage (↑ γH2AX, ↑ 8-OHdG)	[93]
*N. thorellii* x (*ventricosa* x *maxima*)/ethyl acetate fraction from methanol extract	leukemia cell lines (HL-60, K-562, MOLT-4)	↓ viability: IC_50_ 3.85, 3.68, 3.73 μg/mL ↑ apoptosis (↑ subG1, ↑ annexin V, ↑ caspases 3/7) ↑ ROS: ↑ MitoSOX, ↑ MMP ↑ expression of antioxidant genes DNA damage (↑ γH2AX, ↑ G8-OHdG)	[94]

IC_50_—half maximal inhibitory concentration; ROS—reactive oxygen species; GSH-glutathione; MitoSOX—mitochondrial superoxide; MMP—mitochondrial membrane potential; 8-OxodG—8-Oxo-2′-deoxyguanosine; G8-OHdG—8-hydroxy-2′-deoxyguanosine; ↓—decrease; ↑—increase.

**Table 4:** Cytotoxic activity of naphthoquinones isolated from Droseraceae and Nepenthaceae.

Compound	Species	Cytotoxicity/Reference Line	Ref
plumbagin	*N. thorelii* x (*ventricosa* x *maxima*)/aerial parts	leukemia cells (24 h assay) acute promyelocytic (HL-60): IC_50_ = 0.35 µg/mL chronic myelogenous (K-562): IC_50_ = 0.4 µg/mL T-cell acute lymphocytic (MOLT-4): IC_50_ = 0.19 µg/mL	[94]
plumbagin	*N. alata*	human breast cancer (MCF-7): IC_50_ = 3.5 μM (48 h assay) human epithelial ovarian cancer (SK-OV-3): IC_50_ = 13.1 μM (48 h assay)	[119]
plumbagin	*Di. muscipula*	murine lymphocytic leukemia (P388): IC_50_ = 0.01 µg/mL human colon carcinoma (HCT116): IC_50_ = 0.11 µg/mL	[86]
plumbagin	*Di. muscipula*	human breast cancer MDA-MB-468: IC_50_ = 0.4 μM human breast adenocarcinoma (MCF-7): IC_50_ = 1.8 μM/epithelial cell line (MCF-10a)	[117]
plumbagin	*Di. muscipula*	promyelocytic leukemia (HL-60): IC_50_ = 2.7 μM	[75]
3-hydroxymethylplumbagin	*Di. muscipula*	murine lymphocytic leukemia (P388): IC_50_ = 0.08 µg/mL	[86]
hydroplumbagin ß-D-glucopyranoside	*Di. muscipula*	murine lymphocytic leukemia (P388): IC_50_ = 2.0 µg/mL	[86]
3-chloroplumbagin	*Di. muscipula*	human breast cancer (MDA-MB-468): IC_50_ = 0.6 μM human breast adenocarcinoma (MCF-7): IC_50_ = 2 μM/epithelial cell line (MCF-10a)	[117]
ramentaceone	*Dr. aliciae*	human leukemic monocyte lymphoma (U937): IC_50_ = 3.2 µM human cervical cancer (HeLa): IC_50_ = 51 µM human breast adenocarcinoma (MCF-7): IC_50_ = 17 µM human colorectal carcinoma (HCT-116): IC_50_ = 37 µM	[122]
ramentaceone	*Dr. aliciae*	human promyelocytic leukemia (HL-60): IC_50_ = 8.75 μM	[118]
isoshinanolone	*Di. muscipula*	murine lymphocytic leukemia (P388): IC_50_ = 0.4 µg/mL	[86]
diomuscinone	*Di. muscipula*	murine lymphocytic leukemia (P388): IC_50_ = 0.06 µg/mL	[86]

**Table 5:** Anti-inflammatory activity of Droseraceae and Nepenthaceae plants.

Species/Extract	Experimental Model	Effect	Ref
*Dr. rotundifolia*/water (w), 70% ethanol (e)	human neutrophil elastase	Inhibition with IC_50_ 5 (w), 1 (e) μg/mL	[131]
*Dr. madagascariensis*/70% ethanol	human neutrophil elastase	Inhibition with IC_50_ 9.4 μg/mL	[130]
*Dr. rotundifolia*/water, ethanol	HET-CAM assay	500 μg/pellet: inhibition 88% (w), 98% (e) (hydrocortisone 50 μg/pellet: 89%)	[129]
*Dr. madagascariensis*/water, ethanol	HET-CAM assay	500 μg/pellet: inhibition 51% (w), 89% (e) (hydrocortisone 50 μg/pellet: 89%)	[129]
*Dr. burmannii* 70% methanol in water	LPS-induced murine macrophage (RAW 264.7)	30–80 µg/mL ↓ NO, ↓ iNOS, ↓ TNF-α, ↓ COX-2	[87]
*Dr. rotundifolia*, *Dr. tokaiensis/*80% ethanol in water	human mast cells (HMC-1) induced by PMA-activated T-cell membrane	200 µg/mL suppressed morphological changes, expression of inflammatory genes, TNF-α, GZMB, IL1β, and ICAM-1	[133]

HET-CAM assay—hen’s eggs test—chorioallantoic membrane; LPS—lipopolysaccharide; PMA—phorbol 12-myristate 13-acetate; iNOS—inducible nitric oxide synthase; TNF-α—tumor necrosis factor-alpha, COX-2—cyclooxygenase 2; GZMB—Granzyme B; IL1β—interleukine-1; ICAM-1—intracellular adhesion molecule-1.

**Table 6:** Other types of activity of carnivorous Nepenthales plants.

Species/Extract	Activity	Experimental Model	Effect	Ref
*Dr. rotundifolia*/ethanol	impact on respiratory tract	bronchial epithelial cell line (16HBE)	↑ cell viability ca 6.9%, ↑ epidermal growth factors, ↑ proteins related with detoxification, ↑ cytokines	[142]
*Dr. rotundifolia*/water (w), 70 ethanol (e)	antispasmodic	guinea-pig ileum induced with carbachol (M_3_ receptor)	0.5 mg/mL depressed the response to carbachol to 86% (w) and to 64% (e)	[131]
*Dr. madagascariensis*/70% ethanol	antispasmodic	guinea-pig ileum induced with carbachol/histamine/PGF_2á_ (M_3_, H_1_, contractile prostanoid receptors, respectively)	M_3_: 0.5 mg/mL depressed the response to 72%, and 1 mg/mL to 35% H_1_: 0.5 mg/mL depressed the response to 75% PGF_2á_: no effect	[130]
*Dr. rotundifolia*/90% ethanol (e), aqueous fraction (w)	antispasmodic	tracheal slices of C57BL/6N mice	↓ acetylcholine-induced contractions (e,w); ↓ K+ induced contraction (w)	[143]
*Dr. rotundifolia*/90% ethanol, aqueous fraction	ciliary beat frequency	tracheal slices of C57BL/6N mice	↑ciliary beat frequency	[143]
*Dr. rotundifolia, Dr. tokaiensis, Dr. spatulata, Dr. peltata*/80% methanol	antiaging	ribose-gelatin mixture	10,20,50 µg/mL: ↓ formation of CMA and CML	[156]
*Dr. burmannii*/90% ethanol	antiepileptic	mice with pentylenetetrazole-induced seizures	500 mg/kg bw: delaying the onset of convulsions, shortening the duration of seizures	[157]
*Dr. burmannii/*70% methanol	hepatoprotective	iron-overloaded Swiss albino mice	↓ ALAT, ↓ ASAT, ↓ ALP, ↓ bilirubin ↑ SOD, ↑ CAT, ↑ GST, ↑ GSH ↓ liver iron, ↓ serum ferritin ↓ lipid and protein peroxidation ↓ collagen content restoration of healthy liver	[140]
*Dr. burmannii/*90% ethanol (e), water (w)	antifertility	female Wistar rats with normal estrus cycles	250 and 450 mg/kg bw: ↓ implantation to 78.74% and 85.23% (e); ↓ implantation to 67.22% and 83.66% (w)	[161]
*N. thorelii* root/chloroform	antimalarial	*Plasmodium falciparum*	Inhibition of growth: IC_50_ 10 µg/mL	[147]
*T. peltatum* root (r), stem bark (s)/chloroform	antimalarial	*Plasmodium falciparum*	Inhibition of growth: IC_50_ 53 ng/mL (r) IC_50_ 76 (s) ng/mL	[148]
*T. peltatum* root (r), stem bark (s)/chloroform	antimalarial	*Plasmodium berghei*	Inhibition of growth: IC_50_ 103 ng/mL (r) IC_50_ 279 ng/mL (s)	[148]
*N. mirabilis*/methanol	anti-osteoporotic	RANκL-induced murine bone-marrow macrophages (RAW 264.7)	Inhibition of TRAP expression (↓ osteoclast differentiation)	[141]
*N. bicalcarata*/methanol	antidiabetic	diabetic rats	300 mg/kg bw for 6 week: ↓ blood glucose level	[64]
*N. mirabilis*/ethanol (e), water (w)	antidiabetic	enzymatic activity assay	Inhibition of α-glucosidase: IC_50_ = 32.7 (e), 3.3 (w) µg/mL and α –amylase: IC_50_ = 73.6 (e), 296.7 (w) µg/mL	[158]

CMA—Nω-(carboxymethyl)arginine; CML—Nε-(carboxymethyl)lysine, PGF_2á_—prostaglandin F2á, M3—muscarinic receptors, H1—histamine receptors, TRAP—tartrate-resistant acid phosphatase; ↓ decrease; ↑ increase.

## Data Availability

Data are contained within the article.
